# Training in Interdisciplinary, Practice-Oriented Minority Aging Research: Honoring the Work of Dr. James Jackson

**DOI:** 10.1093/geroni/igab046.1176

**Published:** 2021-12-17

**Authors:** Briana Mezuk, Robert Taylor, Roland Thorpe

## Abstract

Few scientists had the breadth and depth of scholarship, the keen interest in interdisciplinary scientific collaboration, and the commitment to mentoring the next generation of scientists as Dr. James Jackson. His passing remains a tremendous loss for the field. This symposium, organized by members of the Michigan Center for Urban African American Aging Research (MCUAAAR), which was founded by James over 20 years ago, reflects on the impact of transdisciplinary team science, of the importance of research networks and resource sharing, of the need to center research within practice and community, and of the scientific innovation that comes from integrating conceptual models, data sources, and methodological approaches from seemingly disparate fields. The session is co-chaired by Dr. Robert Taylor, longtime faculty member and current PI of MCUAAAR. The talk by session chair Dr. Briana Mezuk will discuss the ways in which the training approach of Analysis Core has inspired new training programs on integrative methods focused on minority health and disparities. The talk by Dr. Tam Perry will describe the innovations of the Community Liaison and Recruitment Core, including how COVID-19 impacted the activities of the Healthier Black Elder Center. The third talk by Dr. Rodlescia Sneed, a MCUAAAR early-career scientist, provides an example of how this Center supports interdisciplinary minority aging research through her project focused on older adults who have a history of incarceration. Finally, Discussant Dr. Roland Thorpe, a member of the MCUAAAR Advisory Board, will reflect on Dr. Jackson’s legacy of mentorship and collaboration.

